# Associations between aldehyde dehydrogenase 2 (ALDH2) rs671 genetic polymorphisms, lifestyles and hypertension risk in Chinese Han people

**DOI:** 10.1038/s41598-017-11071-w

**Published:** 2017-09-11

**Authors:** Cong Ma, Bingxiang Yu, Weihua Zhang, Weimin Wang, Liping Zhang, Qiang Zeng

**Affiliations:** Department of International Inpatient, Institute of Health Management, Chinese PLA General Hospital, Beijing, China

## Abstract

Hypertension is a multiple factor disease which was influenced by gene, environment, and lifestyle. Several studies confirmed that the ALDH2 rs671 polymorphism is associated with hypertension. However, the evidence remains inconclusive. Whether lifestyle affects blood pressure in different genotype groups have not been clarified, either. The subjects were adult Chinese Han people who received health examination in the period from December 2014 to December 2015. Detection of the ALDH2 r671 polymorphism was determined by polymerase chain reaction. Lifestyle data were collected using self-administered questionnaires. Basic characteristics and fasting venous blood sample were collected at baseline. 4018 subjects were eligible for participation.The frequencies of the ALDH2 rs671 genotype were 68.67% (GG), 28.67%(GL), 2.66%(LL), respectively. Pepole who harbored the L allele were less likely to develop incident hypertension. There was a significant association between food frequency and hypertension in the L genotype group. Fried food intake was significantly increased the risk of hypertension in the L genotype group. Our study suggested that ALDH2 rs671 L-genotypes are protective factors for hypertension in Han Chinese. Consumption of fried food accelerated the development of hypertension in individuals with poor metabolism of acetaldehyde.

## Introduction

Hypertension is the common and severe cardiovascular disease. It is the main cause of coronary heart disease and myocardial infarction. Longterm hypertension can also lead to stroke, cerebral vascular diseases, congestive heart failures and end-stage renal diseases which increased mortality and shortened life expectancy^1–3^. China is a fast-growing and -ageing nation with approximately 200 million hypertensive patients^4^. Therefore, it is important for prevention and control of hypertension in China.

Hypertension is a multiple factor disease which was influenced by gene, environment, lifestyle, demographic factors, etc^5–7^. To date, numerous studies have been carried out to investigate the association between gene polymorphism and hypertension^8–10^. It was reported that approximately 69% systolic and 51% diastolic blood pressure variation is genetically determined^11^. Thus, susceptibility gene association study plays an important role in the pathogenesis of essential hypertension^12^.

Aldehyde dehydrogenase (ALDH) super family is the principle enzymes responsible for hepatic metabolism of ethanol. ALDH2 primary contribute to the *in vivo* metabolism of acetaldehyde, a direct metabolite of ethanol^13, 14^. Human ALDH2 gene is mapped to chromosome 12q24. A sequence rs671 polymorphism (also named Glu487Lys, or Glu504Lys) has been widely studied. There is a G-to-L missense mutation in which glutamate at position 504 is replaced by lysine, then forms GG, GL and LL three patterns (also indicated as GG, GA and AA or *1/*1, *1/*2 and *2/*2)^15, 16^. Evidence about Glu504Lys polymorphism focused on alcohol-induced“flushing” syndrome including the red face, nausea, and rapid heart rate^17^.

Recently, several studies confirmed that the ALDH2 rs671 polymorphism is associated with hypertension. However, the evidence remains inconclusive. Whether there is a relationship between ALDH2 rs671 polymorphism and hypertension^18–20^, whether the relationship between hypertension and ALDH2 rs671 polymorphism is dependent or independent of alcohol consumption^21, 22^ and sex remain controversial^20, 23, 24^.

In addition to acetaldehyde metabolism, ALDH2 polymorphism plays an a wide range of physiological functions, including the regulation of inflammation, oxidative stress and so on^25–27^. Dietary elements have long been known to play a critical role in the physiological or pathological response to tissue inflammation and oxidative stress which plays an important role in the development of hypertension^28, 29^. For example, a high-fat diet causes damage at the cellular and molecular levels, and it triggers an oxidative stress process^30^. Fish is a source of N-3 polyunsaturated fatty acids which may modify inflammation^31^. Furthermore, dietary fibre from vegetables, fruit and cereals have the potential to mitigate oxidative stress and inflammation^32^. Therefore, the effect may differ among various populations depending on gene-lifestyle interactions. Although lifestyle modifications are essential for the management of hypertension^33–35^, whether lifestyle affects blood pressure in different genotype groups was rarely reported.

We conduct this study in order to derive the comprehensive associations between the aldehyde dehydrogenase polymorphism, lifestyle and the risk of essential hypertension in Chinese Han people. This work is beneficial to individualization of hypertension management.

## Results

Among 4074 recruited patients, 56 patients were excluded: 21 had malignant tumor history, 5 did not sign an informed consent form, 10 had inadequate blood samples, and 20 provided no questionnaire. Therefore, 4018 patients were included in the analysis.

### Baseline characteristics

The frequencies of the ALDH2 genotype were 68.67% (GG), 28.67%(GL), 2.66%(LL), respectively.The mean age of the patients was 48.12 ± 7.99 years and 69.81% of patients were men. There were significant difference in age, sex, body mass index (BMI), waist to hip ratio(WHR), heart rate, history of diabete, stroke, coronary heart disease(CHD), intestinal disease, smoking and drinking habits between hypertensive group and normotensive group (P < 0.05) (Table 1).

**Table 1:** Demographic clinical characteristics of Chinese Han people suffering from hypertension compared to the control group.

Characteristics	Hypertensive group (1181)	Normotensive group(2837)	P-value	OR(95%CI)
Age	50.78 ± 7.00	47.05 ± 8.12	<0.001	1.06(1.05–1.08)
Sex	979(82.90)	1826(64.36)	<0.001	0.37(0.31–0.44)
BMI	27.13 ± 3.11	24.79 ± 3.19	<0.001	1.26(1.23–1.29)
WHR	0.94 ± 0.061	0.89 ± 0.08	<0.001	4.18(3.48–5.02)
HR	74.58 ± 10.66	73.21 ± 9.82	<0.001	1.01(1.00–1.02)
Smoking	402(34.04)	852(30.03)	<0.05	1.35(1.22–1.49)
Drinking	797(67.49)	1462(57.53)	<0.001	1.47(1.32–1.64)
Stoke	21(1.79)	17(0.60)	<0.001	3.00(1.58–5.71)
Diabetes	267(22.61)	252(8.89)	<0.001	3.00(2.48–3.61)
CHD	112(9.50)	84(2.96)	<0.001	3.44(2.57–4.61)
Gastric disease	468(39.63)	1059(37.34)	0.174	1.10(0.96–1.27)
Intestinal disease	309(26.16)	599(21.11)	<0.001	1.32(1.13–1.55)
Chronic hepatitis	60(5.08)	169(5.96)	0.275	0.85(0.62–1.14)

Abbreviations: BMI, body mass index; WHR, waist to hip ratio; HR, heart rate; CHD, coronary heart disease; CI, confidence interval; OR, odds ratio; Continuous variables were expressed as means ± standard deviations when normally distributed and categorical variables were expressed as percentages. OR and P value were obtained from a univariate logistic regression analysis based on Chinese Han people who were normotensive or hypertensive in Health Management Institute (n = 4018).

Each sample was assigned the genotype and the frequency of each genotype and allele was calculated in the patients and control group. The genotype and allele frequencies in the cases and controls were checked for deviation from Hardy-Weinberg quilibrium. No deviation was observed (Table 2).

**Table 2:** Distribution of Genotypes of ALDH2 rs671 follows Hardy-Weinberg equilibrium (HWE) among Chinese Han people with hypertension group and controls.

genotype	hypertension Cases	HWE P value	Controls	HWE P value
GG	871	P = 0.066	1888	P = 0.66
GL	295		857	
LL	15		92	

P value > 0.05 indicates that the studied population was in HWE.

### The relevant factors of essential hypertension

Table 3 shows the results of multivariate logistic regression analysis, the significant variables identified as risk factors of hypertension were age, sex, BMI, heart rate, diabetes, coronary heart disease, gastric disease. Notably, It showed that those carrying GL or LL genotype were at a lower risk of hypertension(OR = 0.76, 95%CI:0.61–0.96, P = 0.03). No significant association was found between lifestyle and hypertension.

**Table 3:** Odds ratios and 95% confidence intervals for ALDH2 rs671 polymorphisms, lifestyle and base characteristics in relation to risk of hypertension.

	P-value	OR(95%CI)		P-value	OR(95%CI)
Age	<0.001	1.07(1.05–1.09)	ALDH2rs671 (L allele carriers)	0.03	0.76(0.61–0.96)
Sex	0.03	0.59(0.36–0.96)	Stoke	0.56	1.35(0.49–3.71)
BMI	<0.001	1.23(1.17–1.29)	Diabetes	<0.001	1.75 1.32–2.32)
WHR	0.94	1.15(0.03–39.59)	CHD	0.003	1.91(1.25–2.92)
HR	<0.001	1.02(1.01–1.03)	Gastric disease	0.04	1.26(1.01–1.57)
Smoking	0.74	1.03(0.88–1.20)	Intestinal disease	0.15	1.20(0.94–1.53)
Drinking	0.06	1.20 (0.10–1.45)	Chronic hepatitis	0.63	1.11(0.72–1.71)
Cereals	0.53		Meat	0.19	
seldom		1(ref)	seldom		1(ref)
1 to 2 times	0.35	1.59(0.60–4.20)	1 to 2 times	0.87	0.95(0.54–1.69)
3 to 4 times	0.40	1.53(0.57–4.07)	3 to 4 times	0.57	0.85(0.49–1.49)
5 to 7 times	0.22	1.79(0.70–4.6)	5 to 7 times	0.22	0.70 (0.41–1.23)
Fishes	0.63		Fresh fruit and vegetables	0.10	
seldom		1(ref)	seldom		1(ref)
1 to 2 times	0.24	0.83(0.60–1.14)	1 to 2 times	0.15	0.54 (0.24–1.25)
3 to 4 times	0.44	0.88(0.63–1.23)	3 to 4 times	0.34	0.67 (0.29–1.53)
5 to 7 times	0.25	0.81(0.56–1.17)	5 to 7 times	0.10	0.51(0.22–1.14)
Milk and products	0.52		Eggs	0.56	
seldom		1(ref)	seldom		1(ref)
1 to 2 times	0.47	0.91(0.69–1.18)	1 to 2 times	0.75	1.08(0.67–1.73)
3 to 4 times	0.17	0.81(0.60–1.10)	3 to 4 times	0.80	0.94(0.59–1.51)
5 to 7 times	0.26	0.84(0.62–1.14)	5 to 7 times	0.62	1.13(0.71–1.79)
Bean products	0.53		Desserts	0.06	
seldom		1(ref)	seldom		1(ref)
1 to 2 times	0.87	0.97(0.65–1.44)	1 to 2 times	0.01	0.72(0.55–0.93)
3 to 4 times	0.45	1.17(0.78–1.75)	3 to 4 times	0.17	0.80(0.57–1.10)
5 to 7 times	0.73	1.08(0.70–1.67)	5 to 7 times	0.09	0.71(0.47–1.06)
Fried foods	0.39		Salted and smoked foods	0.90	
seldom		1(ref)	seldom		1(ref)
1 to 2 times	0.21	1.18(0.91–1.52)	1 to 2 times	0.73	1.05(0.81–1.34)
3 to 4 times	0.19	1.27(0.89–1.82)	3 to 4 times	0.54	1.11(0.80–1.52)
5 to 7 times	0.19	1.38(0.85–2.24)	5 to 7 times	0.85	0.96(0.64–1.44)

Abbreviations: BMI, body mass index; WHR, waist to hip ratio; HR, heart rate; CHD, coronary heart disease; CI, confidence interval; OR, odds ratio; Food intake was divided into 4 categories, seldom eat, 1 to 2 times a week, 3 to 4 times a week, 5 to 7 times a week with seldom eat as the reference category. Meat contains Pork, beef, mutton, poultry. Data were obtained from a multivariable logistic regression analysis based on Chinese Han people with complete covariable data who were normotensive or hypertensive in Health Management Institute (n = 4018).

### Comparison of the effects of lifestle in essential hypertension between the G genotype and the L genotype groups in Chinese Han people

In multivariate logistic regression analysis, the risk factors of essential hypertension in G genetype of ALDH2 rs671 were similar as in L genetype genetypes, However, the influence of lifestyle on hypertension were obviously different in the two groups. No significant association was found between lifestyle and hypertensive risk in G genotype group. On the contrary, there was a significant association between food frequency and hypertension in the L genotype group. The fried food intake is a risk factor for the development of hypertension in the L genotype group(GL + LL vs GG, OR = 1.70,95% CI:1.03–2.82, P = 0.04 for 1–2 times fried food per week intake; OR = 2.09, 95% CI:1.04–4.22, P = 0.04 for 3–4 times fried food per week intake; OR = 3.53, 95% CI:1.29–9.67, P = 0.01 for 5–7 times desserts per week intake) (Table 4).

**Table 4:** Comparison of the relationship between lifestyle and essential hypertension in the G genotype and the L genotype groups for Chinese Han people.

	GG genetype		GL + LL genetype	
	P	OR(95%CI)	P	OR(95%CI)
Age	<0.001	1.06(1.04–1.09)	<0.001	1.10(1.01–1.14)
BMI	<0.001	1.22(0.73–1.66)	<0.001	1.25 (1.15–1.37)
HR	0.035	1.01(1.00–1.03)	0.001	1.03(1.01–1.06)
Diabetes	<0.001	2.00(1.43–2.80)	0.46	1.25(0.69–2.27)
CHD	0.001	2.38 (1.40–4.06)	0.56	1.28(0.57–2.88)
Gastric disease	0.31	1.15 (0.88–1.49)	0.009	1.76(1.15–2.69)
Fried foods	1.00		0.04	
seldom		1(ref)		1(ref)
1 to 2times a week	0.87	1.03(0.76–1.39)	0.04	1.70 (1.03–2.82)
3 to 4 times a week	0.89	1.03 (0.60–1.64)	0.04	2.09 (1.04–4.22)
5 to 7 times a week	0.80	1.08(0.61–1.99)	0.01	3.53 (1.29–9.67)

Abbreviations:CI, confidence interval; OR, odds ratio; Food intake was divided into 4 categories, seldom eat, 1 to 2times a week, 3 to 4 times a week, 5 to 7 times a week with seldom eat as the reference category. Data were obtained from a multivariable logistic regression analysis based on Chinese Han people with complete covariable data who were normotensive or hypertensive in Health Management Institute (n = 4018).

### Association between ALDH2 genotypes and the blood lipid, blood glucose, hepatic and renal function

Subjects of GG genotypes had higher serum total cholesterol(TC) than subjects of GL and LL genotype (4.73 vs.4.66, P = 0.02), the same as triglyceride(TG)(1.86 vs.1.67, P = 0.001) and high density lipoprotein-cholesterol (HDL-C) (1.22 vs.1.19, P = 0.005). There were significant difference of fasting blood glucose(FBG) and postprandial blood glucose(PBG) level between the two groups. In GG genotypes, the FBG(5.65 vs. 5.50, P = 0.012) and PBG(7.48 vs.7.27, P = 0.006) were higher than in GL and LL geneotypes. Compared to GG genotypes, the levels of blood urea nitrogen(4.91 vs.5.05, P = 0.001), serum creatinine (67.49 vs. 69.55, P = 0.001) were increased and uric acid (346.67 vs.340.72, P = 0.04) were decreased markedly in GL and LL genotype. Those with GG geneotype exhibited significantly higher levels than those with GL and LL geneotypes for three primary parameters of hepatic function, AST(21.59 vs.19.90, P = 0.001), ALT(26.65 vs.22.29, P < 0.001) and γ-GT(45.68 vs.32.13, P < 0.001) (Table 5).

**Table 5:** Comparison of the blood lipid, blood glucose, hepatic and renal function between the G genotype and the L genotype groups in Chinese Han people.

	GG genotype groups	GLand LL genotype groups	P value
TC	4.73 ± 0.92	4.66 ± 0.87	0.02
LDL	3.11 ± 0.83	3.11 ± 0.80	0.96
TG	1.86 ± 0.49	1.67 ± 1.18	<0.001
HDL	1.22 ± 0.34	1.19 ± 0.35	0.005
FBG	5.65 ± 1.44	5.50 ± 1.39	0.012
PBG	7.48 ± 2.51	7.27 ± 2.37	0.006
BUN	4.91 ± 1.47	5.05 ± 1.20	0.001
UA	346.67 ± 88.66	340.72 ± 82.04	0.040
SCr	67.49 ± 22.51	69.55 ± 16.31	0.001
γ-GT	45.68 ± 48.60	32.13 ± 32.56	<0.001
ALT	25.65 ± 20.00	22.29 ± 15.32	<0.001
AST	21.59 ± 15.71	19.90 ± 13.95	0.001
TBA	3.96 ± 4.99	4.06 ± 8.40	0.682
TB	11.67 ± 5.41	10.89 ± 4.59	<0.001
TP	70.42 ± 5.23	69.72 ± 5.24	<0.001
DB	3.89 ± 1.56	3.67 ± 1.41	<0.001

Continuous variables were expressed as means ± standard deviations when normally distributed. Abbreviations and unit:TC, total cholesterol, mmol/L; LDL-C, low-density lipoprotein cholesterol, mmol/L; TG: triglycerides, mmol/L; HDL-C, high-density lipoprotein cholesterol, mmol/L; FBG, fasting blood glucose, mmol/L; PBG, postprandial blood glucose, mmol/L; BUN, blood urea nitrogen, umol/L; γ-GT, γ-glutamyl-transferase, U/L; UA,uric acid, umol/L; SCr creatinine, umol/L; ALT, alanine aminotransferase, U/L; AST, aspartate aminotransferase, U/L; TBA, total bile acid, umol/L; TB, total bilirubin, umol/L;TP, total protein, umol/L; DB, direct bilirubi, umol/L.

## Discussion

Mitochondrial ALDH2 is an enzyme responsible for metabolizing toxic aldehydes. Studies have shown that ALDH2 is a protective factor against oxidative stress, ALDH2 deficiency increases oxidative stress which is the predisposing factors of hypertension^36^. Acetaldehyde and lipid aldehyde can cause atherosclerosis through the formation of protein byproducts, and ALDH2 play a role in anti-atherosclerosis via its detoxication. Heterozygous ALDH2 mutant (GLtype) has only 10% to 45% individual enzyme activity, while the activity of homozygous (LL type) individual is only 1%~5% activity^37^.

Previous studies demonstrated that the frequency of ALDH2 G allele differed significantly among different races, even among different East Asian populations^38, 39^. Previous studies showed that the ALDH2 genetic polymorphism plays an important role in several pathological conditions, including hepatitis, certain types of carcinomas, coronary artery disease, cerebral infarction^40–42^.

Recent few studies suggested that this polymorphism is probably associated with hypertension. However, this association is uncertain. In addition, it was hypothesized that lifestyle may contribute to the individual variation in blood pressure in different ALDH2 genetype, but it was rarely studied. Our study confirmed these conclusions and suspicions.

Theoretically, decreased ALDH2 mutant enzyme activity may lead to an increase in reactive oxygen which predisposed to hypertension. Interestingly, several studies suggested that the rs671 L genotype was a protective factor in hypertension development. Wu Y^43^ conducted case-control study and meta-analysis and indicated a decreased risk of hypertensive risk in AA/AG genotype. Zhang SY^18^ found that the rs671 GG genotype was associated with an increased risk of hypertension compared with the AG/AA genotypes, especially in men and this association was independent of alcohol consumption. OtaM^19^ reported that blood pressure in the *1/*2 or *2/*2 group were significantly lower than those in the *1/*1 group. The amount of daily alcohol intake was a predict factor of systolic blood pressure in participants who harbored the ALDH2 genetic polymorphism *1/*2 or *2/2*. However, in the study of Amamoto K, the blood pressure and ALDH2 gene polymorphisms were not associated after the correction of the confounding factors^20^.

In this study, SBP and DBP were significantly lower in participants who harbored the ALDH2 genetic polymorphism GL or LL than in those who harbored the ALDH2 genetic polymorphism GG. Multivariate regression showed pepole who harbored the L allele were less likely to develop incident hypertension.This result is consistent with the findings of OtaM and Zhang SY. The relevance remained after adjustment for age, sex, BMI, heart rate, smoking, drinking, dietary habits, diabetes, coronary heart disease and other comorbidities.

It is generally accepted that alcohol consumption is a potent risk factor for high blood pressure. However, It is not clear whether the effects of alcohol intake on blood pressure differ with the ALDH2 genotype. Previous studies have indicated that the different ALDH2 rs671 genotype could not influence the alcohol-BP relationship in Japanese men^21, 23^. However, studies have shown that the alcohol-BP relationship was significantly stronger in men with wild ALDH2 rs671 genotype than in men with mutation homozygous genotypes in Japanese rural population^22^. Our study was conducted to investigate whether ALDH2 genetic polymorphism and alcohol intake affect the onset of hypertension. After adjusting for the effects of age, body mass index and lifestyle which are strong determinants of hypertension, no relations was found between alcohol intake and hypertension. This results may explain why the ALDH2 rs671 deficiency groups have low enzyme activity but less risk of hypertension. Therefore, we conclude that the rs671 polymorphism may influence the risk of hypertension by some other mechanism independent of alcohol consumption.

It had been postulated that there are differences in genetic factors for hypertension between men and women. Large cohort studies^23^ showed that ALDH2 Glu/Glu genotype is a potential risk factor for hypertension in men, rather than in women. Hui^24^ obeserved that ALDH2 Glu/Glu genotype was the independent risk factor for the Japanese, especially in male patients with hypertension. Amamoto^20^ found that wild ALDH2 genotype correlates with the susceptibility of essential hypertension in Chinese men and ALDH2 deficiency does not affect women’s blood pressure. In our study, when adjust for potential confounding factors, sex is irrelevant to hypertension neither in inactive ALDH2 group nor active ALDH2 group. It had been postulated that gender factors do not affect ALDH2 rs671 polymorphism–related hypertension.

In our study, we investigate whether ALDH2 rs671 polymorphism affects blood pressure through the lifestyle. We found fried food is a risk factor of hypertension in the L genotype group. This effect was not seen in the G genotype group. Moreover, the prevalence of hypertension was raised with increasing consumption of fried food in the L genotype group. We can concluded that consumption of fried food accelerated the development of hypertension in individuals with poor metabolism of acetaldehyde. L genotype group should especially avoid fried food. Its mechanism may be that high-fat diet induced inflammation and oxidative stress in poor metabolism of acetaldehyde individuals. The findings suggested that the lifestyle intervention may be more effective for improvement the prevalence of hypertension in the L genotype group.

In this study, we found that AST, ALT, γ-GT, TB, DB, TC, TG, HDL, FBG, PBG, UA and alcohol intake were significantly higher in the ALDH2 GG genotype group than in the ALDH2 GL and LL genotype group. Meanwhile, Cr, BUN were significantly lower in the ALDH2 GG genotype group than in the ALDH2 GL and LL genotype group. We conclude these changes are related to alcohol consumption. Hao G^42^ reported that alcohol consumption was associated with increased levels of TC and HDL in both genders. Shen Z^44^ suggested that daily alcohol intake was closely associated with hypertriglyceridemia. Sakai Y^45^ reported alcohol use was positively associated with IFG, IGT, and type 2 diabetes mellitus. The mechanism is potentially that alcohol use caused a defect in the secretion pancreas, thereby decreasing in glucose-stimulated insulin secretion. Noborisaka Y^46^ noted alcohol consumption was a significant but weak contributor to decrease the levels of serum creatinine and Ccr increasing. Chung FM^47^ oberserved chronic alcohol consumption stimulates the estimated GFR and CCr. Lieber *et al*.^48^ observed an increase in serum uric acid following acute intoxication in non-gouty alcoholics. Hyun Choi and colleagues^49^ clearly show that alcohol intake is strongly associated with heightened risk of gout in men.

The limitations of our study are the following. First, for the purpose of this study, we combined the ALDH2 heterozygous GL genetype and homozygous LL genetype as one group. Because the ALDH2 homozygous LL genetype sample size was small, the statistical power was low when the participants were divided into three groups according to the ALDH2 genotypes. Second, we only studied chinese Han population, the result does not represent all the population.Therefore, studies with larger sample size are needed to confirm these results and should be testified in different populations worldwide.

In conclusion, our results provides evidence that the ALDH2 rs671 L-genotypes are protective factors for hypertension in Han Chinese, independent of alcohol consumption and sex. The interaction between lifestyle and ALDH2 genetic polymorphism might affect hypertension incidence in adult Chinese Han population. Considering the limitations mentioned, further studies are needed to confirm our findings.

## Methods

### Subjects

Patients who were older than 18 years and received health examination in Health Management Institute of PLA General Hospital during December 2014 to December 2015 were eligible to participate in this cross-sectional study. Patients were excluded if they had the following conditions: secondary hypertension, malignant tumor, acute myocardial infarction, unstable angina, acute phase of stroke, peripheral artery disease, uncontrolled diabetes mellitus (HbA1c >9.0%), or severe liver, kidney, and thyroid dysfunction.

All subjects gave informed consent to participate in the study. After the patient had been seated quietly for 5 min, blood pressure was measured three times with a electronic sphygmomanometer and the average was recorded. Hypertension was diagnosed according to WHO criteria: systolic blood pressure (SBP) ≥140 mmHg and/or diastolic blood pressure (DBP) ≥90 mmHg in three separate times, including those who received pharmacologic treatment with antihypertensive drugs in the preceding two weeks. The normotensive individuals were selected based on SBP <140 mmHg and/or DBP <90 mmHg, excluding those with antihypertensive medication history.

### Detection of ALDH2 Genetypes

Venous blood samples were drawn and collected in ethylenediaminetetraacetic acid (EDTA) tubes, and DNA was extracted by using a whole-blood DNA extraction and purification kit (BaiO, Shanghai, China) according to the manufacturer’s instructions. Samples were coded to allow blinding of the investigators who carried out the genotyping. The ALDH2 genotype was determined by PCR-Genotyping microarray analysis of the three gene type including two important allelic variants (ALDH2*1 and ALDH2*2) using BaiO gene detection kit (Genotyping microarray method, BaiO, Shanghai, China).

### Biochemical indicators

An12-h fasting blood sample was collected for the measurement of hemoglobin A1c, fasting plasma glucose, blood lipids, hepatic function and renal function.

### Evaluation of exposures

All participants were asked to provide information by a self-administrated questionnaire at the time of the first visit and before any diagnostic procedures. The questionnaire included items on sociodemographic characteristics, family and individual medical history, height and weight, smoking and drinking habits. Information on dietary habits was collected through a food frequency questionnaire that included 10 single food items with frequencies in 4 categories.

### Statistical analysis

The means and standard deviations (SD) were calculated for normally distributed continuous variables, and percentages were calculated for categorical variables. Student’s t-test or one way-ANOVA were used for continuous variables and chi-square test or Fisher exact test were used for the categorical variables; Binary logistic regression analysis was performed to determine independent clinical risk factors for hypertension in the stepwise forward selection procedure. Student’s t-test, one way-ANOVA analyses, chi-square test, fisher exact test and binary logistic regression were performed using SPSS version 24.0. Hardy-Weinberg equilibrium (HWE) analysis were investigated by using SNPStats (available online at http://bioinfo.iconcologia.net/SNPstats). All analyses were performed with 95% confidence intervals, and p-values of less than 0.05 were considered statistically significant.

### Study approval

All the methods were performed in accordance with relevant guidelines and regulations and all participants gave a written informed consent to the study protocol prior to inclusion in the study. This study was approved by the ethical commitees of Chinese PLA General Hospital.
